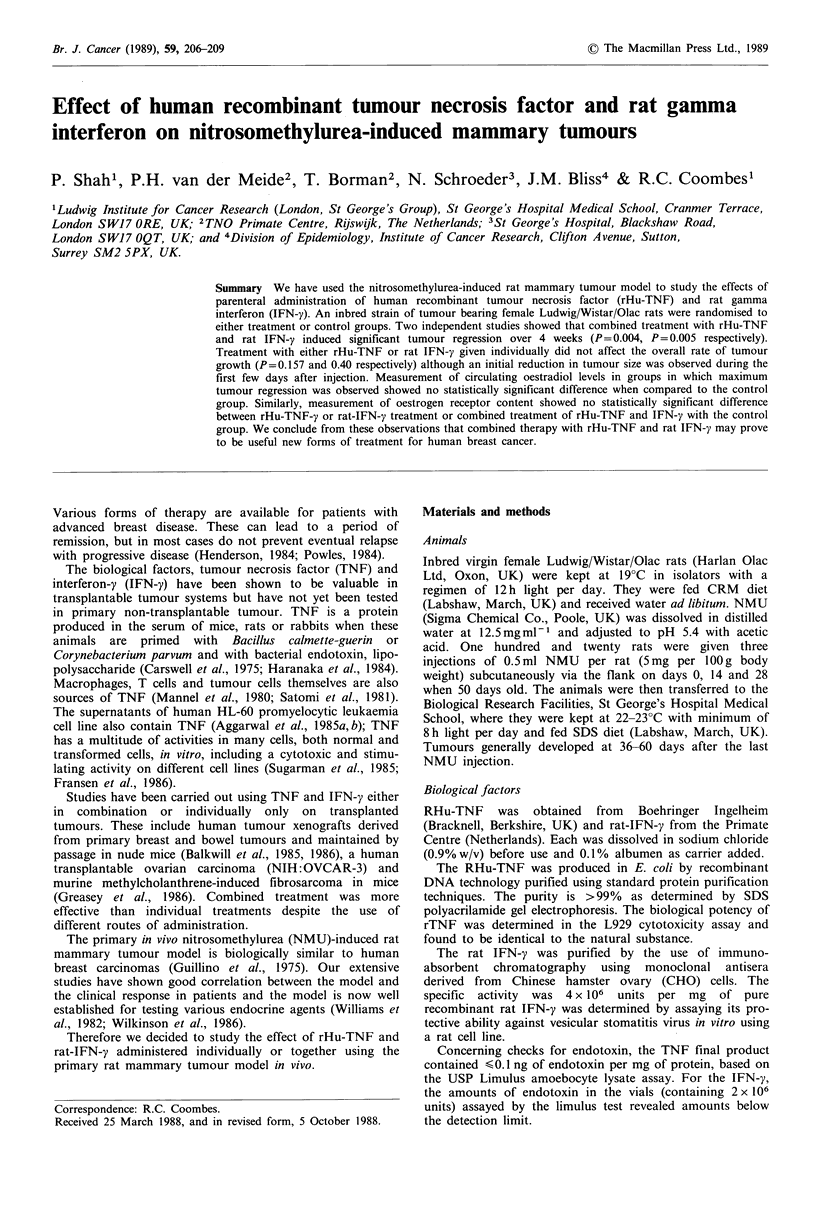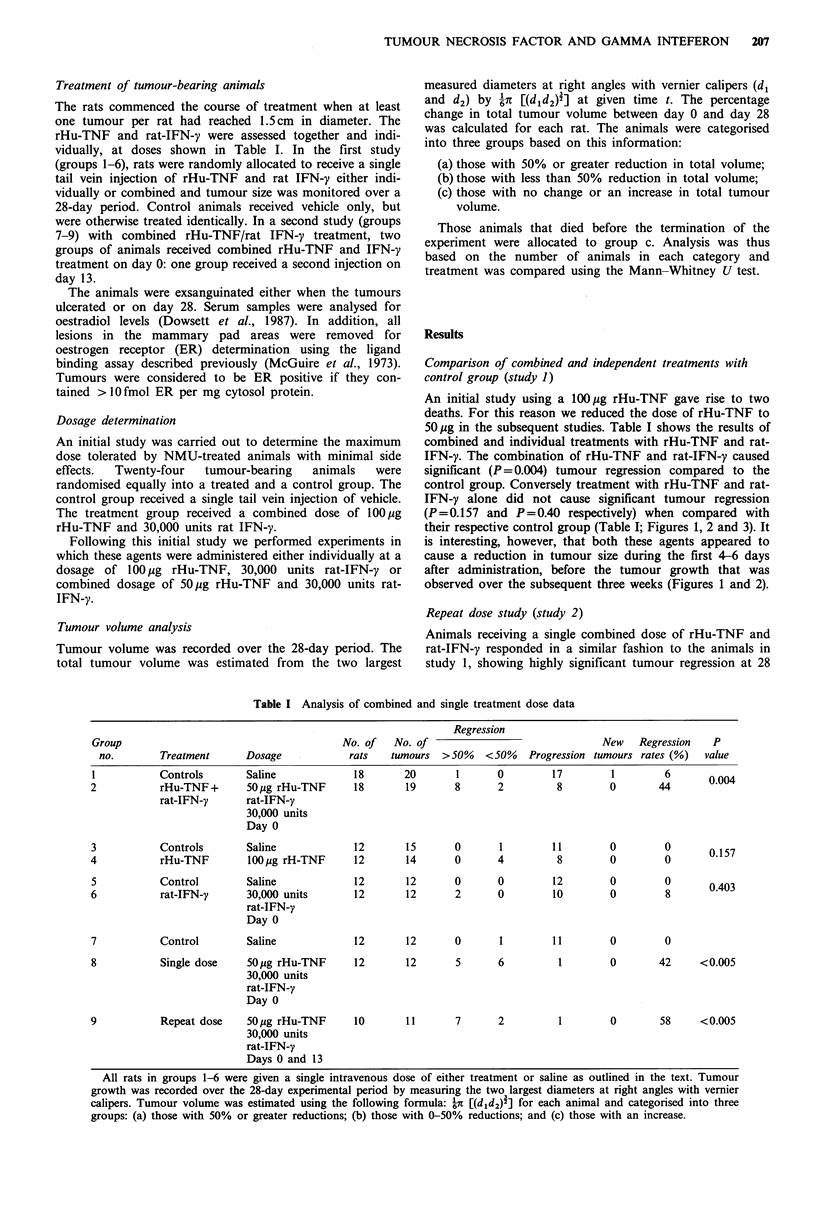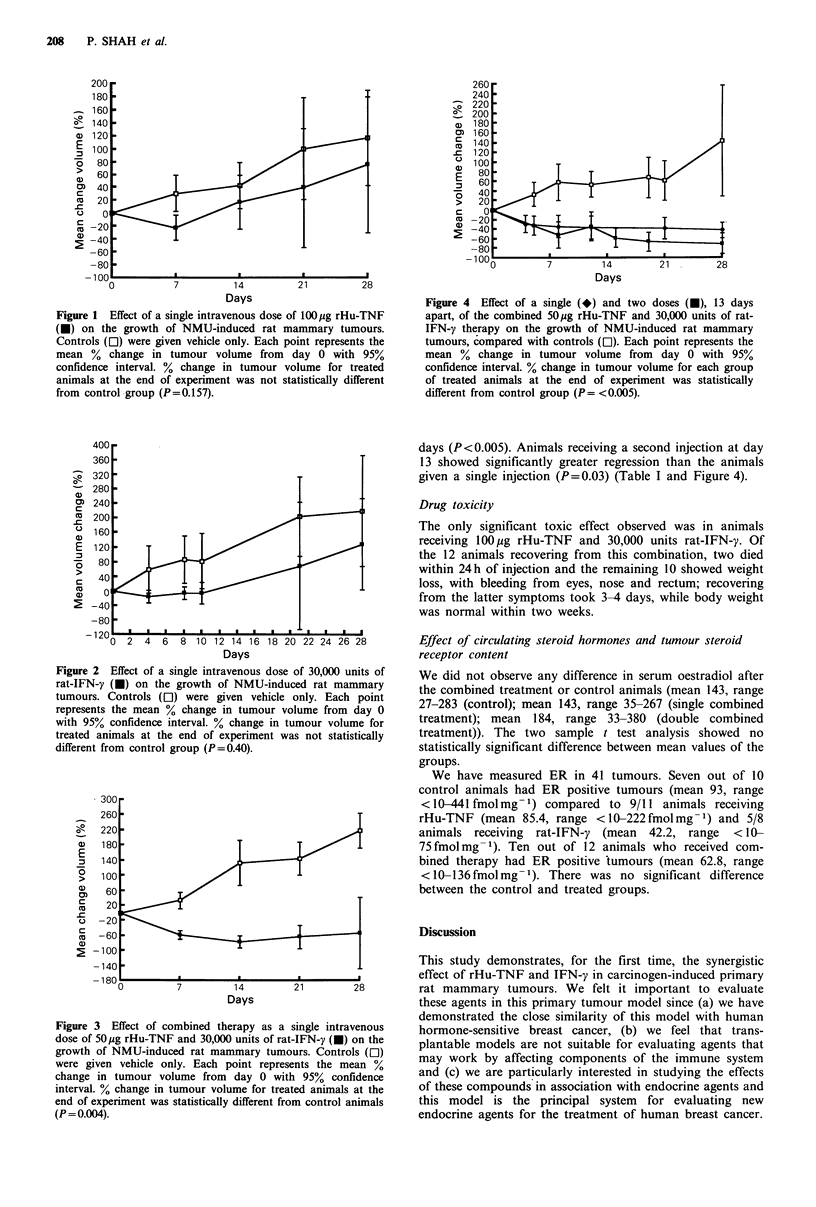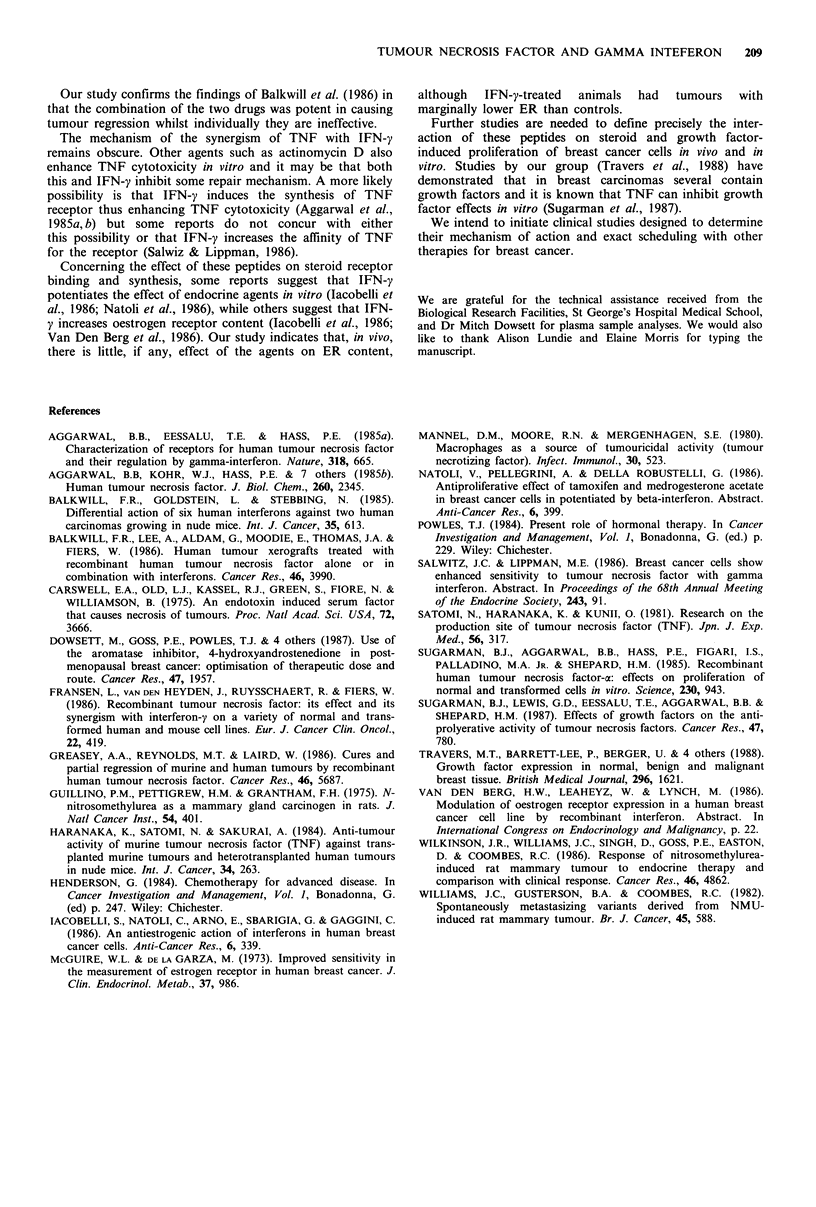# Effect of human recombinant tumour necrosis factor and rat gamma interferon on nitrosomethylurea-induced mammary tumours.

**DOI:** 10.1038/bjc.1989.42

**Published:** 1989-02

**Authors:** P. Shah, P. H. van der Meide, T. Borman, N. Schroeder, J. M. Bliss, R. C. Coombes

**Affiliations:** Ludwig Institute for Cancer Research, St George's Hospital Medical School, London, UK.

## Abstract

We have used the nitrosomethylurea-induced rat mammary tumour model to study the effects of parenteral administration of human recombinant tumour necrosis factor (rHu-TNF) and rat gamma interferon (IFN-gamma). An inbred strain of tumour bearing female Ludwig/Wistar/Olac rats were randomised to either treatment or control groups. Two independent studies showed that combined treatment with rHu-TNF and rat IFN-gamma induced significant tumour regression over 4 weeks (P = 0.004, P = 0.005 respectively). Treatment with either rHu-TNF or rat IFN-gamma given individually did not affect the overall rate of tumour growth (P = 0.157 and 0.40 respectively) although an initial reduction in tumour size was observed during the first few days after injection. Measurement of circulating oestradiol levels in groups in which maximum tumour regression was observed showed no statistically significant difference when compared to the control group. Similarly, measurement of oestrogen receptor content showed no statistically significant difference between rHu-TNF-gamma or rat-IFN-gamma treatment or combined treatment of rHu-TNF and IFN-gamma with the control group. We conclude from these observations that combined therapy with rHu-TNF and rat IFN-gamma may prove to be useful new forms of treatment for human breast cancer.


*Br. J. Cancer* (1989), **59**, 206–209 © The Macmillan Press Ltd., 1989

### Effect of human recombinant tumour necrosis factor and rat gamma interferon on nitrosomethylurea-induced mammary tumours

P. Shah[1], P.H. van der Meide[2], T. Borman[2], N. Schroeder[3], J.M. Bliss[4] & R.C. Coombes[1]

[1]*Ludwig Institute for Cancer Research (London, St George's Group), St George's Hospital Medical School, Cranmer Terrace, London SW17 0RE, UK;* [2]*TNO Primate Centre, Rijswijk, The Netherlands;* [3]*St George's Hospital, Blackshaw Road, London SW17 0QT, UK; and* [4]*Division of Epidemiology, Institute of Cancer Research, Clifton Avenue, Sutton, Surrey SM2 5PX, UK.*

**Summary** We have used the nitrosomethylurea-induced rat mammary tumour model to study the effects of parenteral administration of human recombinant tumour necrosis factor (rHu-TNF) and rat gamma interferon (IFN-γ). An inbred strain of tumour bearing female Ludwig/Wistar/Olac rats were randomised to either treatment or control groups. Two independent studies showed that combined treatment with rHu-TNF and rat IFN-γ induced significant tumour regression over 4 weeks ($P=0.004$, $P=0.005$ respectively). Treatment with either rHu-TNF or rat IFN-γ given individually did not affect the overall rate of tumour growth ($P=0.157$ and 0.40 respectively) although an initial reduction in tumour size was observed during the first few days after injection. Measurement of circulating oestradiol levels in groups in which maximum tumour regression was observed showed no statistically significant difference when compared to the control group. Similarly, measurement of oestrogen receptor content showed no statistically significant difference between rHu-TNF-γ or rat-IFN-γ treatment or combined treatment of rHu-TNF and IFN-γ with the control group. We conclude from these observations that combined therapy with rHu-TNF and rat IFN-γ may prove to be useful new forms of treatment for human breast cancer.

Various forms of therapy are available for patients with advanced breast disease. These can lead to a period of remission, but in most cases do not prevent eventual relapse with progressive disease (Henderson, 1984; Powles, 1984).

The biological factors, tumour necrosis factor (TNF) and interferon-γ (IFN-γ) have been shown to be valuable in transplantable tumour systems but have not yet been tested in primary non-transplantable tumour. TNF is a protein produced in the serum of mice, rats or rabbits when these animals are primed with *Bacillus calmette-guerin* or *Corynebacterium parvum* and with bacterial endotoxin, lipopolysaccharide (Carswell *et al.*, 1975; Haranaka *et al.*, 1984). Macrophages, T cells and tumour cells themselves are also sources of TNF (Mannel *et al.*, 1980; Satomi *et al.*, 1981). The supernatants of human HL-60 promyelocytic leukaemia cell line also contain TNF (Aggarwal *et al.*, 1985*a,b*); TNF has a multitude of activities in many cells, both normal and transformed cells, *in vitro*, including a cytotoxic and stimulating activity on different cell lines (Sugarman *et al.*, 1985; Fransen *et al.*, 1986).

Studies have been carried out using TNF and IFN-γ either in combination or individually only on transplanted tumours. These include human tumour xenografts derived from primary breast and bowel tumours and maintained by passage in nude mice (Balkwill *et al.*, 1985, 1986), a human transplantable ovarian carcinoma (NIH:OVCAR-3) and murine methylcholanthrene-induced fibrosarcoma in mice (Greasey *et al.*, 1986). Combined treatment was more effective than individual treatments despite the use of different routes of administration.

The primary *in vivo* nitrosomethylurea (NMU)-induced rat mammary tumour model is biologically similar to human breast carcinomas (Guillino *et al.*, 1975). Our extensive studies have shown good correlation between the model and the clinical response in patients and the model is now well established for testing various endocrine agents (Williams *et al.*, 1982; Wilkinson *et al.*, 1986).

Therefore we decided to study the effect of rHu-TNF and rat-IFN-γ administered individually or together using the primary rat mammary tumour model *in vivo*.

Correspondence: R.C. Coombes.
Received 25 March 1988, and in revised form, 5 October 1988.

### Materials and methods

### *Animals*

Inbred virgin female Ludwig/Wistar/Olac rats (Harlan Olac Ltd, Oxon, UK) were kept at 19°C in isolators with a regimen of 12 h light per day. They were fed CRM diet (Labshaw, March, UK) and received water *ad libitum*. NMU (Sigma Chemical Co., Poole, UK) was dissolved in distilled water at $12.5\,mg\,ml^{-1}$ and adjusted to pH 5.4 with acetic acid. One hundred and twenty rats were given three injections of 0.5 ml NMU per rat (5 mg per 100 g body weight) subcutaneously via the flank on days 0, 14 and 28 when 50 days old. The animals were then transferred to the Biological Research Facilities, St George's Hospital Medical School, where they were kept at 22–23°C with minimum of 8 h light per day and fed SDS diet (Labshaw, March, UK). Tumours generally developed at 36–60 days after the last NMU injection.

### *Biological factors*

RHu-TNF was obtained from Boehringer Ingelheim (Bracknell, Berkshire, UK) and rat-IFN-γ from the Primate Centre (Netherlands). Each was dissolved in sodium chloride (0.9% w/v) before use and 0.1% albumen as carrier added.

The RHu-TNF was produced in *E. coli* by recombinant DNA technology purified using standard protein purification techniques. The purity is >99% as determined by SDS polyacrilamide gel electrophoresis. The biological potency of rTNF was determined in the L929 cytotoxicity assay and found to be identical to the natural substance.

The rat IFN-γ was purified by the use of immunoabsorbent chromatography using monoclonal antisera derived from Chinese hamster ovary (CHO) cells. The specific activity was $4\times10^6$ units per mg of pure recombinant rat IFN-γ was determined by assaying its protective ability against vesicular stomatitis virus *in vitro* using a rat cell line.

Concerning checks for endotoxin, the TNF final product contained ≤0.1 ng of endotoxin per mg of protein, based on the USP Limulus amoebocyte lysate assay. For the IFN-γ, the amounts of endotoxin in the vials (containing $2\times10^6$ units) assayed by the limulus test revealed amounts below the detection limit.

*Treatment of tumour-bearing animals*

The rats commenced the course of treatment when at least one tumour per rat had reached 1.5 cm in diameter. The rHu-TNF and rat-IFN-γ were assessed together and individually, at doses shown in Table I. In the first study (groups 1–6), rats were randomly allocated to receive a single tail vein injection of rHu-TNF and rat IFN-γ either individually or combined and tumour size was monitored over a 28-day period. Control animals received vehicle only, but were otherwise treated identically. In a second study (groups 7–9) with combined rHu-TNF/rat IFN-γ treatment, two groups of animals received combined rHu-TNF and IFN-γ treatment on day 0: one group received a second injection on day 13.

The animals were exsanguinated either when the tumours ulcerated or on day 28. Serum samples were analysed for oestradiol levels (Dowsett *et al*., 1987). In addition, all lesions in the mammary pad areas were removed for oestrogen receptor (ER) determination using the ligand binding assay described previously (McGuire *et al*., 1973). Tumours were considered to be ER positive if they contained >10 fmol ER per mg cytosol protein.

*Dosage determination*

An initial study was carried out to determine the maximum dose tolerated by NMU-treated animals with minimal side effects. Twenty-four tumour-bearing animals were randomised equally into a treated and a control group. The control group received a single tail vein injection of vehicle. The treatment group received a combined dose of 100 μg rHu-TNF and 30,000 units rat IFN-γ.

Following this initial study we performed experiments in which these agents were administered either individually at a dosage of 100 μg rHu-TNF, 30,000 units rat-IFN-γ or combined dosage of 50 μg rHu-TNF and 30,000 units rat-IFN-γ.

*Tumour volume analysis*

Tumour volume was recorded over the 28-day period. The total tumour volume was estimated from the two largest measured diameters at right angles with vernier calipers ($d_1$ and $d_2$) by $\frac{1}{6}\pi\ [(d_1 d_2)^{\frac{3}{2}}]$ at given time *t*. The percentage change in total tumour volume between day 0 and day 28 was calculated for each rat. The animals were categorised into three groups based on this information:

(a) those with 50% or greater reduction in total volume;
(b) those with less than 50% reduction in total volume;
(c) those with no change or an increase in total tumour volume.

Those animals that died before the termination of the experiment were allocated to group c. Analysis was thus based on the number of animals in each category and treatment was compared using the Mann–Whitney *U* test.

### Results

*Comparison of combined and independent treatments with control group (study 1)*

An initial study using a 100 μg rHu-TNF gave rise to two deaths. For this reason we reduced the dose of rHu-TNF to 50 μg in the subsequent studies. Table I shows the results of combined and individual treatments with rHu-TNF and rat-IFN-γ. The combination of rHu-TNF and rat-IFN-γ caused significant ($P = 0.004$) tumour regression compared to the control group. Conversely treatment with rHu-TNF and rat-IFN-γ alone did not cause significant tumour regression ($P = 0.157$ and $P = 0.40$ respectively) when compared with their respective control group (Table I; Figures 1, 2 and 3). It is interesting, however, that both these agents appeared to cause a reduction in tumour size during the first 4–6 days after administration, before the tumour growth that was observed over the subsequent three weeks (Figures 1 and 2).

*Repeat dose study (study 2)*

Animals receiving a single combined dose of rHu-TNF and rat-IFN-γ responded in a similar fashion to the animals in study 1, showing highly significant tumour regression at 28

**Table I** Analysis of combined and single treatment dose data

| *Group no.* | *Treatment* | *Dosage* | *No. of rats* | *No. of tumours* | *Regression* >50% | *Regression* <50% | *Progression* | *New tumours* | *Regression rates (%)* | *P value* |
|---|---|---|---|---|---|---|---|---|---|---|
| 1 | Controls | Saline | 18 | 20 | 1 | 0 | 17 | 1 | 6 | 0.004 |
| 2 | rHu-TNF + rat-IFN-γ | 50 μg rHu-TNF rat-IFN-γ 30,000 units Day 0 | 18 | 19 | 8 | 2 | 8 | 0 | 44 | |
| 3 | Controls | Saline | 12 | 15 | 0 | 1 | 11 | 0 | 0 | 0.157 |
| 4 | rHu-TNF | 100 μg rH-TNF | 12 | 14 | 0 | 4 | 8 | 0 | 0 | |
| 5 | Control | Saline | 12 | 12 | 0 | 0 | 12 | 0 | 0 | 0.403 |
| 6 | rat-IFN-γ | 30,000 units rat-IFN-γ Day 0 | 12 | 12 | 2 | 0 | 10 | 0 | 8 | |
| 7 | Control | Saline | 12 | 12 | 0 | 1 | 11 | 0 | 0 | |
| 8 | Single dose | 50 μg rHu-TNF 30,000 units rat-IFN-γ Day 0 | 12 | 12 | 5 | 6 | 1 | 0 | 42 | <0.005 |
| 9 | Repeat dose | 50 μg rHu-TNF 30,000 units rat-IFN-γ Days 0 and 13 | 10 | 11 | 7 | 2 | 1 | 0 | 58 | <0.005 |

All rats in groups 1–6 were given a single intravenous dose of either treatment or saline as outlined in the text. Tumour growth was recorded over the 28-day experimental period by measuring the two largest diameters at right angles with vernier calipers. Tumour volume was estimated using the following formula: $\frac{1}{6}\pi\ [(d_1 d_2)^{\frac{3}{2}}]$ for each animal and categorised into three groups: (a) those with 50% or greater reductions; (b) those with 0–50% reductions; and (c) those with an increase.

**Figure 1** Effect of a single intravenous dose of 100 μg rHu-TNF (■) on the growth of NMU-induced rat mammary tumours. Controls (□) were given vehicle only. Each point represents the mean % change in tumour volume from day 0 with 95% confidence interval. % change in tumour volume for treated animals at the end of experiment was not statistically different from control group ($P = 0.157$).

**Figure 4** Effect of a single (◆) and two doses (■), 13 days apart, of the combined 50 μg rHu-TNF and 30,000 units of rat-IFN-γ therapy on the growth of NMU-induced rat mammary tumours, compared with controls (□). Each point represents the mean % change in tumour volume from day 0 with 95% confidence interval. % change in tumour volume for each group of treated animals at the end of experiment was statistically different from control group ($P = < 0.005$).

**Figure 2** Effect of a single intravenous dose of 30,000 units of rat-IFN-γ (■) on the growth of NMU-induced rat mammary tumours. Controls (□) were given vehicle only. Each point represents the mean % change in tumour volume from day 0 with 95% confidence interval. % change in tumour volume for treated animals at the end of experiment was not statistically different from control group ($P = 0.40$).

**Figure 3** Effect of combined therapy as a single intravenous dose of 50 μg rHu-TNF and 30,000 units of rat-IFN-γ (■) on the growth of NMU-induced rat mammary tumours. Controls (□) were given vehicle only. Each point represents the mean % change in tumour volume from day 0 with 95% confidence interval. % change in tumour volume for treated animals at the end of experiment was statistically different from control animals ($P = 0.004$).

days ($P < 0.005$). Animals receiving a second injection at day 13 showed significantly greater regression than the animals given a single injection ($P = 0.03$) (Table I and Figure 4).

### *Drug toxicity*

The only significant toxic effect observed was in animals receiving 100 μg rHu-TNF and 30,000 units rat-IFN-γ. Of the 12 animals recovering from this combination, two died within 24 h of injection and the remaining 10 showed weight loss, with bleeding from eyes, nose and rectum; recovering from the latter symptoms took 3–4 days, while body weight was normal within two weeks.

### *Effect of circulating steroid hormones and tumour steroid receptor content*

We did not observe any difference in serum oestradiol after the combined treatment or control animals (mean 143, range 27–283 (control); mean 143, range 35–267 (single combined treatment); mean 184, range 33–380 (double combined treatment)). The two sample *t* test analysis showed no statistically significant difference between mean values of the groups.

We have measured ER in 41 tumours. Seven out of 10 control animals had ER positive tumours (mean 93, range <10–441 fmol mg$^{-1}$) compared to 9/11 animals receiving rHu-TNF (mean 85.4, range <10–222 fmol mg$^{-1}$) and 5/8 animals receiving rat-IFN-γ (mean 42.2, range <10–75 fmol mg$^{-1}$). Ten out of 12 animals who received combined therapy had ER positive tumours (mean 62.8, range <10–136 fmol mg$^{-1}$). There was no significant difference between the control and treated groups.

### Discussion

This study demonstrates, for the first time, the synergistic effect of rHu-TNF and IFN-γ in carcinogen-induced primary rat mammary tumours. We felt it important to evaluate these agents in this primary tumour model since (a) we have demonstrated the close similarity of this model with human hormone-sensitive breast cancer, (b) we feel that transplantable models are not suitable for evaluating agents that may work by affecting components of the immune system and (c) we are particularly interested in studying the effects of these compounds in association with endocrine agents and this model is the principal system for evaluating new endocrine agents for the treatment of human breast cancer.

Our study confirms the findings of Balkwill *et al.* (1986) in that the combination of the two drugs was potent in causing tumour regression whilst individually they are ineffective.

The mechanism of the synergism of TNF with IFN-$\gamma$ remains obscure. Other agents such as actinomycin D also enhance TNF cytotoxicity *in vitro* and it may be that both this and IFN-$\gamma$ inhibit some repair mechanism. A more likely possibility is that IFN-$\gamma$ induces the synthesis of TNF receptor thus enhancing TNF cytotoxicity (Aggarwal *et al.*, 1985*a, b*) but some reports do not concur with either this possibility or that IFN-$\gamma$ increases the affinity of TNF for the receptor (Salwiz & Lippman, 1986).

Concerning the effect of these peptides on steroid receptor binding and synthesis, some reports suggest that IFN-$\gamma$ potentiates the effect of endocrine agents *in vitro* (Iacobelli *et al.*, 1986; Natoli *et al.*, 1986), while others suggest that IFN-$\gamma$ increases oestrogen receptor content (Iacobelli *et al.*, 1986; Van Den Berg *et al.*, 1986). Our study indicates that, *in vivo*, there is little, if any, effect of the agents on ER content, although IFN-$\gamma$-treated animals had tumours with marginally lower ER than controls.

Further studies are needed to define precisely the interaction of these peptides on steroid and growth factor-induced proliferation of breast cancer cells *in vivo* and *in vitro*. Studies by our group (Travers *et al.*, 1988) have demonstrated that in breast carcinomas several contain growth factors and it is known that TNF can inhibit growth factor effects *in vitro* (Sugarman *et al.*, 1987).

We intend to initiate clinical studies designed to determine their mechanism of action and exact scheduling with other therapies for breast cancer.

We are grateful for the technical assistance received from the Biological Research Facilities, St George's Hospital Medical School, and Dr Mitch Dowsett for plasma sample analyses. We would also like to thank Alison Lundie and Elaine Morris for typing the manuscript.

**References**

AGGARWAL, B.B., EESSALU, T.E. & HASS, P.E. (1985*a*). Characterization of receptors for human tumour necrosis factor and their regulation by gamma-interferon. *Nature*, **318**, 665.

AGGARWAL, B.B, KOHR, W.J., HASS, P.E. & 7 others (1985*b*). Human tumour necrosis factor. *J. Biol. Chem.*, **260**, 2345.

BALKWILL, F.R., GOLDSTEIN, L. & STEBBING, N. (1985). Differential action of six human interferons against two human carcinomas growing in nude mice. *Int. J. Cancer*, **35**, 613.

BALKWILL, F.R., LEE, A., ALDAM, G., MOODIE, E., THOMAS, J.A. & FIERS, W. (1986). Human tumour xerografts treated with recombinant human tumour necrosis factor alone or in combination with interferons. *Cancer Res.*, **46**, 3990.

CARSWELL, E.A., OLD, L.J., KASSEL, R.J., GREEN, S., FIORE, N. & WILLIAMSON, B. (1975). An endotoxin induced serum factor that causes necrosis of tumours. *Proc. Natl Acad. Sci. USA*, **72**, 3666.

DOWSETT, M., GOSS, P.E., POWLES, T.J. & 4 others (1987). Use of the aromatase inhibitor, 4-hydroxyandrostenedione in post-menopausal breast cancer: optimisation of therapeutic dose and route. *Cancer Res.*, **47**, 1957.

FRANSEN, L., VAN DEN HEYDEN, J., RUYSSCHAERT, R. & FIERS, W. (1986). Recombinant tumour necrosis factor: its effect and its synergism with interferon-$\gamma$ on a variety of normal and transformed human and mouse cell lines. *Eur. J. Cancer Clin. Oncol.*, **22**, 419.

GREASEY, A.A., REYNOLDS, M.T. & LAIRD, W. (1986). Cures and partial regression of murine and human tumours by recombinant human tumour necrosis factor. *Cancer Res.*, **46**, 5687.

GUILLINO, P.M., PETTIGREW, H.M. & GRANTHAM, F.H. (1975). *N*-nitrosomethylurea as a mammary gland carcinogen in rats. *J. Natl Cancer Inst.*, **54**, 401.

HARANAKA, K., SATOMI, N. & SAKURAI, A. (1984). Anti-tumour activity of murine tumour necrosis factor (TNF) against transplanted murine tumours and heterotransplanted human tumours in nude mice. *Int. J. Cancer*, **34**, 263.

HENDERSON, G. (1984). Chemotherapy for advanced disease. In *Cancer Investigation and Management, Vol. 1*, Bonadonna, G. (ed) p. 247. Wiley: Chichester.

IACOBELLI, S., NATOLI, C., ARNO, E., SBARIGIA, G. & GAGGINI, C. (1986). An antiestrogenic action of interferons in human breast cancer cells. *Anti-Cancer Res.*, **6**, 339.

McGUIRE, W.L. & DE LA GARZA, M. (1973). Improved sensitivity in the measurement of estrogen receptor in human breast cancer. *J. Clin. Endocrinol. Metab.*, **37**, 986.

MANNEL, D.M., MOORE, R.N. & MERGENHAGEN, S.E. (1980). Macrophages as a source of tumouricidal activity (tumour necrotizing factor). *Infect. Immunol.*, **30**, 523.

NATOLI, V., PELLEGRINI, A. & DELLA ROBUSTELLI, G. (1986). Antiproliferative effect of tamoxifen and medrogesterone acetate in breast cancer cells in potentiated by beta-interferon. Abstract. *Anti-Cancer Res.*, **6**, 399.

POWLES, T.J. (1984). Present role of hormonal therapy. In *Cancer Investigation and Management, Vol. 1*, Bonadonna, G. (ed.) p. 229. Wiley: Chichester.

SALWITZ, J.C. & LIPPMAN, M.E. (1986). Breast cancer cells show enhanced sensitivity to tumour necrosis factor with gamma interferon. Abstract. In *Proceedings of the 68th Annual Meeting of the Endocrine Society*, **243**, 91.

SATOMI, N., HARANAKA, K. & KUNII, O. (1981). Research on the production site of tumour necrosis factor (TNF). *Jpn. J. Exp. Med.*, **56**, 317.

SUGARMAN, B.J., AGGARWAL, B.B., HASS, P.E., FIGARI, I.S., PALLADINO, M.A. JR. & SHEPARD, H.M. (1985). Recombinant human tumour necrosis factor-$\alpha$: effects on proliferation of normal and transformed cells *in vitro*. *Science*, **230**, 943.

SUGARMAN, B.J., LEWIS, G.D., EESSALU, T.E., AGGARWAL, B.B. & SHEPARD, H.M. (1987). Effects of growth factors on the anti-prolyerative activity of tumour necrosis factors. *Cancer Res.*, **47**, 780.

TRAVERS, M.T., BARRETT-LEE, P., BERGER, U. & 4 others (1988). Growth factor expression in normal, benign and malignant breast tissue. *British Medical Journal*, **296**, 1621.

VAN DEN BERG, H.W., LEAHEYZ, W. & LYNCH, M. (1986). Modulation of oestrogen receptor expression in a human breast cancer cell line by recombinant interferon. Abstract. In *International Congress on Endocrinology and Malignancy*, p. 22.

WILKINSON, J.R., WILLIAMS, J.C., SINGH, D., GOSS, P.E., EASTON, D. & COOMBES, R.C. (1986). Response of nitrosomethylurea-induced rat mammary tumour to endocrine therapy and comparison with clinical response. *Cancer Res.*, **46**, 4862.

WILLIAMS, J.C., GUSTERSON, B.A. & COOMBES, R.C. (1982). Spontaneously metastasizing variants derived from NMU-induced rat mammary tumour. *Br. J. Cancer*, **45**, 588.